# Predictive Factors and Nomogram for 30-Day Mortality in Heatstroke Patients: A Retrospective Cohort Study

**DOI:** 10.5811/westjem.23666

**Published:** 2025-03-22

**Authors:** Anxin Li, Yuchen Zhang, Xiaoshi Zhang, Zixiao Duan, Yan Chen, Xiaoyan Jiang, Wuquan Deng

**Affiliations:** * Chongqing University Central Hospital, Chongqing Emergency Medical Centre, School of Medicine, Department of Endocrinology, Chongqing, People’s Republic of China; † Chongqing Medical University the Second Affiliated Hospital, Department of Hematology, Chongqing, People’s Republic of China

## Abstract

**Objective:**

Heatstroke (HS) is a severe condition associated with significant morbidity and mortality. In this study we aimed to identify early risk factors that impacted the 30-day mortality of HS patients and establish a predictive model to assist clinicians in identifying the risk of death.

**Methods:**

We conducted a retrospective cohort study, analyzing the clinical data of 203 HS patients between May 2016–September 2024. The patients were divided into two groups: those who had died within 30 days of symptom onset; and those who had survived. We analyzed the risk factors affecting 30-day mortality. A nomogram was drawn to visualize the clinical model. We used the receiver operating characteristic (ROC) curve and calibration curve to verify the accuracy of the nomogram. A decision curve analysis was also performed to evaluate the clinical usefulness of the nomogram.

**Results:**

Within a 30-day period, 57 patients (28.08%) died. The APACHE II score, the ratio of lactate-to-albumin (LAR), and the core temperature at 30 minutes after admission were independent risk factors for death of HS patients at 30 days. The area under the ROC curve (AUC) for predicting mortality based on the APACHE II score was 0.867, with a sensitivity of 96.5% and a specificity of 61.6%. Moreover, the AUC for predicting mortality based on the LAR was 0.874, with a sensitivity of 93.0% and a specificity of 77.4%. The AUC based on the core temperature at 30 minutes after admission was 0.774, with a sensitivity of 70.2% and a specificity of 78.8%. Finally, the AUC for predicting death due to HS using the combination of these three factors was 0.928, with a sensitivity of 82.5% and a specificity of 91.8%. The calibration curve and the decision-curve analysis showed that the new nomogram had better accuracy and potential application value in predicting the prognosis of HS patients.

**Conclusion:**

A nomogram with these three indicators in combination—APACHE II score, lactate-to-albumin ratio, and core temperature at 30 minutes after admission—can be used to predict 30-day mortality of heatstroke patients.

## INTRODUCTION

Heatstroke (HS) is a severe condition associated with significant morbidity and mortality. The clinical syndrome characteristics of HS include the body’s inability to regulate temperature due to exposure to high temperatures and/or intense physical activity, resulting in an elevation of core temperature and potentially leading to a life-threatening systemic disorder.^1^,^2^ Misset et al reported that the incidence rate of classic HS during summer heat waves in France is 17.6-26.5/100,000.^3^ There is no large-scale HS epidemiology data in the People’s Republic of China to date, since China’s territory is vast and the climate varies significantly across different regions. In some areas such as the city mentioned in this study, the highest temperature in summer can maintain above 39°C (102.2°F).

The treatment for HS patients involves controlling body temperature and improving heated-induced organ injuries.^4^ It has been found that aggressive treatment can improve the prognosis of patients with mild HS. The in-hospital mortality rate of HS patients was 5% in the United States (US), and patients’ race/ethnicity makes no difference in mortality.^2^ The mortality rate of exertional HS (EHS) among youth in the US was <5%.^5^,^6^ The crude mortality rate from HS in

Saudi Arabia is 50%. while the mortality rate of HS in other desert climate countries was lower at 5.6%.^8^ Severe HS still carries a significant mortality rate, with rates exceeding 40%.^9^ This risk is even higher in elderly individuals, where mortality rates can reach up to 50%.^10^ Therefore, it is crucial for clinicians to identify HS patients at high risk of death based on early indicators.

There has been some research on the independent risk factors and predictive model for prognosis of HS.^11^–^14^ Zhong et al^11^ and Wu et al^12^ focused on the risk factors and prediction of mortality from HS. However, their studies concentrated only on male patients. Shao et al^13^ and Wei et al^14^ also constructed nomograms for predicting survival in HS patients. Nevertheless, their studies were focused solely on the elderly. Hence, it is plausible to suggest that their studies exhibit a potential selection bias. There is no consensus on assessing mortality risk in HS patients during the early stages.

In this study, we looked at HS patients who were admitted to Chongqing Emergency Medical Centre and the Second Affiliated Hospital of Chongqing Medical University. After strictly enforcing the inclusion and exclusion criteria, adult patients 19–89 years of age were enrolled, among whom males accounted for 63.1%. We conducted this retrospective cohort research to analyze data from an eight-year period. Our analyses focused on the clinical characteristics, risk factors, and establishment of a predictive model to facilitate the prediction of mortality in HS patients. The Acute Physiology and Chronic Health Evaluation II (APACHE II) has been extensively validated in intensive care unit (ICU) patients, as a highly reliable prognostic scoring system. Its simplicity, clinical utility, accuracy, and validity make it even more reliable. APACHE-IV was first implemented in 2006, based on data collected from ICU patients in the US,^15^ but there have been no reports on its application to HS patients in other countries. We used APACHE II to evaluate the severity and prognosis of HS patients in this study.

## METHODS

### Subjects’ Inclusion and Exclusion Criteria

This study included adult patients who were admitted to the hospitals between May 2016–September 2024 and met the diagnostic criteria for HS as defined by the Chinese Expert Consensus on the Diagnosis and Treatment of HS,^16^ considering that HS can cause damage to multiple organs and systems. The inclusion criteria were as follows: 1) medical history information: exposure to high temperature or high humidity environments or engaging in high-intensity exercise; and 2) clinical presentations (at least one of the following four): central nervous system dysfunction (such as coma, convulsions, delirium, abnormal behavior); core body temperature exceeding 40 °C; functional impairment of multiple organs (at least two) (such as liver, kidney, striated muscle and gastrointestinal tract); severe coagulopathy; or disseminated intravascular coagulation (DIC). The exclusion criteria were as follows: 1) congenital coagulopathy; 2) severe chronic liver or kidney disease; 3) malignant tumors; 4) septic shock; 5) acute severe viral myocarditis; and 6) thyroid storm. The combination of these diseases increases the patient’s risk of death. All patients received Basic Life Support based on their condition, and targeted cooling and organ function support were provided when necessary.

**Population Health Research Capsule**

What do we already know about this issue?

*Heatstroke (HS) is associated with significant morbidity and mortality. It is crucial for clinicians to identify HS patients at high risk of death based on early indicators.*

What was the research question?

*The nomogram in this study improved the accuracy of the 30-day death-risk assessment for HS.*

What was the major finding of the study?

*The area under receiver operating characteristic curve for predicting death due to HS was 0.928 (95% CI 0.889–0.968, P <0.001) with a sensitivity of 82.5% and a specificity of 91.8%.*

How does this improve population health?

*Considering that HS can cause multiorgan functional disturbance, this nomogram can be used to identify the patient’s risk of death and improve the prognosis.*

This study was conducted at Chongqing Emergency Medical Centre and the Second Affiliated Hospital of Chongqing Medical University. The former was the primary research unit, the Ethics Committee of which approved the research protocol. Since this study did not involve patient intervention measures, informed consent was waived by the hospital’s ethics committee (RS202416).

### Data Collection

We collected baseline data from electronic health records, including age, sex, basic diseases, and the condition of the basic diseases prior to the onset of HS through their past medical history. Other recorded baseline data included core temperature (rectal temperature), heart rate (HR), mean arterial pressure (MAP), respiratory rate (RR), Glasgow Coma Scale (GCS) scores,^17^ and APACHE II scores^18^ at the time of admission. The cooling time, which refers to the time taken to cool the body to a core temperature below 38.5°C, was also recorded.^19^ The core temperature measurements were recorded at 30 minutes, two hours, and three hours after admission.

We collected clinical and laboratory data related to organ function, including white blood cells (WBC) and platelets, hemoglobin levels, levels of high-sensitivity C-reactive, alanine transaminase (ALT), aspartate amino transferase, albumin, creatine kinase (CK), myohemoglobin, troponin I, urea nitrogen, creatinine (Cr), blood glucose (BG), and lactic acid. Routine coagulation indicators were recorded, including prothrombin time, activated partial thrombin time, fibrinogen, and D-dimer. and the lactate-to albumin ratio (LAR), calculated with the values of lactate and albumin. The hospital length of stay (LOS) and hospitalization fees were also documented.

Patient records and other information were anonymized and de-identified before analysis.

### Statistical Analysis

We used SPSS Statistics 27.0 package (IBM Corp, Armonk, NY) for data analysis. Continuous variables are presented as mean values with their respective minimum and maximum ranges, or as mean ± standard deviation. Count and rank data were standardized and reported as medians and interquartile ranges. To compare count data among multiple independent samples we used the Kruskal-Wallis H test and the nonparametric Mann-Whitney U test to compare count data among multiple independent samples and two sets of measurement data, respectively. Statistical significance was determined by a *P*-value <0.05. A Cox regression model was established with the occurrence of 30-day death caused by HS as the dependent variables, with 17 indicators as the independent variables. Indicators already included in the APACHE II were not analyzed separately. We used a stepwise method to screen independent variables to identify which indicators had an impact on the 30-day prognosis of HS patients.

Risk factors were subsequently included in the multifactor Cox regression model. We developed a nomogram based on logistic regression to assess the impact of independent risk factors on clinical prognosis significance. The logistic regression model was established with the “lrm” function in the rms package R 4.2.1 language (R Foundation for Statistical Computing, Vienna, Austria), and we used the “plot” function to draw the nomogram. The ROC curve, calibration curve, and decision curve were used to evaluate the accuracy and clinical prediction efficiency of the nomogram.

## RESULTS

### General Information of Enrolled Patients

**Figure 1:**
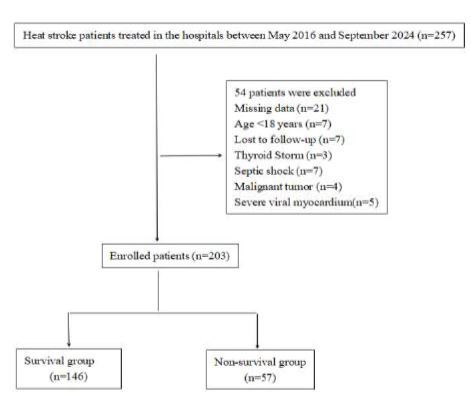
Flow chart of patient enrollment.

Among of the initial group of 257 patients with HS, some were excluded during the enrollment process. Specifically, 21 patients were screened out due to the absence of clinical data or insufficient quantifiable results. We also excluded seven patients who were <18 years of age. Furthermore, seven patients who were transferred to another facility before the completion of initial evaluation were lost to follow-up. It is worth noting that three patients experienced a hyperthyroidism crisis, while seven patients developed septic shock. Moreover, four patients were diagnosed with malignant tumors, and five others had severe viral myocarditis (Figure 1). Among 203 patients, there were 57 non-survivors (28.08%). We found no significant differences between the non-survivor and the survivor groups in terms of sex distribution, age, the time from onset to treatment, and underlying diseases. Among enrolled patients, 102 patients had classic heat stroke (CHS), while 101 patients had exertional HS; the non-survivor group had a higher proportion of CHS compared to the survivor group. The average time from onset to treatment was 8.29 hours, with a range of 0.5–73 hours (Table 1).

The average core temperature at admission was 39.09±1.77°C, with the highest core temperature recorded at 42.0°C. The non-survivor group had higher core temperatures within three hours of admission compared to the survivor group. The average cooling time was 3.13 hours. There were no significant differences in cooling time between the two groups, which was inconsistent with a previous study,^7^ perhaps due to the death of some patients before their core temperature reached 38.5°C in our study. The mean HR was 118.5±31.51 beats per minute (min), the mean RR was 25.04±6.60 breaths/min, and the MAP was 82.87±21.62 millimeters of mercury mmHg. The patients who died had higher HR, RR, and lower MAP than those of survivors (Table 1).

The non-survivor group also showed more severe damage to renal function at admission, with higher levels of Cr compared to the survivor group. There were no significant differences between the two groups in terms of liver function, coagulation, cardiovascular system damage, and respiratory function. The non-survivors did not have increased levels of WBC, platelets, MB, Hs-CRP, and BG, and had increased levels of lactate, Cr, the LAR, and higher APACHE II score. The APACHE II scores including the GCS scores in the non-survivor group were higher than those in the survivor group. The GCS scores in the survivor group were higher than the non-survivor group in this xstudy, which was consistent with a previous study.^20^ The GCS score alone is insufficient for assessing the condition because it is an assessment of the level of consciousness and does not reflect other neurological manifestations such as transient convulsions, which occurred in some patients prior to admission.^21^

The average LOS was 6.20 days, and there was no significant difference between the two groups. The mean hospitalization fee was $2,354.32 USD, and the hospitalization cost was higher in the non-survivor group compared to the survivor group.

### Predictive Factors and Nomogram for 30-Day Mortality in Heatstroke Patients

#### Predictive Factors in HS Patients’ Mortality

**Figure 2:**
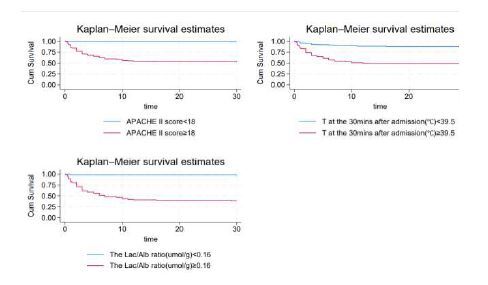
Kaplan-Meier analysis to examine the association of independent risk factors with the risk of death from heatstroke. *APACHE-II*, Acute Physiology and Chronic Health Evaluation**;** *Lac/Alb*, lactate to albumin**;** *T*, core temperature.

The results showed that the independent risk factors affecting the mortality of HS patients were the core temperature at 30 minutes after admission, the APACHE II score, and the LAR. Each unit of increase in core temperature at 30 minutes after admission, APACHE II score, and LAR was associated with a 1.639-fold, 1.102-fold, 12.772-fold increased risk of death in patients of HS, respectively (Table 2). To verify the association between independent risk factors and the risk of death caused by HS, the patients were divided into two groups based on APACHE II scores, LARs, and core temperatures at 30 minutes after admission. The difference in the 30-day mortality based on different APACHE II scores also showed a statistically significant difference (chi square 53.85, *P* < 0.001) (Figure 2). There was a significant difference in 30-day mortality between patients with a LAR ≥0.160 and those with a LAR <0.160 (chi square 91.32, *P* < 0.001) (Figure 2). The difference in the 30-day mortality based on different core temperatures at 30 minutes after admission was statistically significant (chi square 39.09, *P* < 0.001).

#### Nomogram for 30-Day Mortality in Heatstroke Patients

Since these three indicators were identified as independent prognostic factors, they were combined to develop a predictive model for 30-day mortality. The formula for the predictive model was:


$$\begin{array}{l} \text{Y}=-27.681+0.155\times \text{APACHE II score}+5.143\times \text{the}\\ \text{LAR}\;(\text{micromoles per gram})+0.559\times \text{T}\;\text{at}\;30\;\text{minutes}\\ \text{after admission }(\text{core temperature}{}^{\circ}\text{C}) \end{array}$$

**Figure 3:**
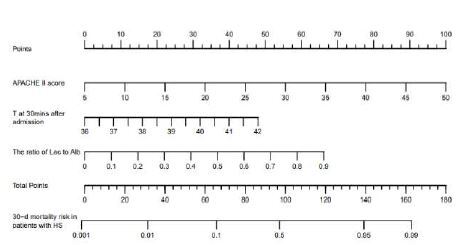
Nomogram model for predicting 30-day mortality risk in patients with heatstroke. *APACHE-II*, Acute Physiology and Chronic Health Evaluation**;** *HS*, heatstroke; *Lac/Alb*, lactate to albumin**;** *T*, core temperature.

Each variable in the nomogram has a corresponding score for a line segment. By calculating the total score of each variable for each patient, the probability of predicting the patient’s death at 30 days can be obtained (Figure 3). If a patient had an APACHE II score of 15, a core temperature of 39°C at 30 minutes after admission, and a LAR of 0.5, according to the nomogram, the values would be 22 points, 24 points, and 34 points, respectively. So, 80 points in total would be obtained, which corresponds to a mortality risk of 96%. In this way, clinicians could quickly assess that this patient has a 96% risk of dying within 30 days and to plan treatment and monitoring accordingly. The nomogram provides an important foundation for clinical decision-making, enabling clinicians to make more precise judgments in complex situations.

The AUC for predicting mortality due to HS based on the core temperature at 30 minutes after admission was 0.774 (95% confidence interval [CI] 0.694–0.854, *P*<0.001). The optimal cut-off value for this indicator was 39.5°C, with a sensitivity of 70.2% and a specificity of 78.8% (Table 3). The AUC for predicting mortality based on the APACHE II was 0.867 (95% CI 0.817–0.916, *P*<0.001), indicating a good predictive accuracy. The optimal cut-off value for this indicator was 18, with a sensitivity of 96.5% and a specificity of 61.6% (Table 3). The AUC for predicting mortality based on the LAR was 0.874 (95% CI 0.827–0.921, *P*<0.001), indicating a high predictive accuracy. The optimal cut-off value for this indicator was 0.160, with a sensitivity of 93.0% and a specificity of 77.4% (Table 3).

**Figure 4:**
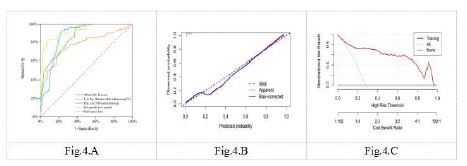
ROC curve, calibration curve, and decision curve of the nomogram. *APACHE-II*, Acute Physiology and Chronic Health Evaluation**;** *Lac/Alb*, lactate to albumin**;** *T*, core temperature.

The AUC for predicting mortality based on the predictive model combining all three indicators was 0.928 (95% CI 0.889–0.968, *P* < 0.001), indicating a high predictive accuracy. The sensitivity of the model was 82.5% and the specificity was 91.8% (Table 3, Figure 4A). The results of calibration curve analysis showed that the probability of death of HS patients at 30 days predicted by the nomogram was very close to the actual probability (Figure 4B), indicating high accuracy of the nomogram. Decision curve analysis showed that the model had a high net benefit value over the entire threshold probability (Figure 4C). Furthermore, the ROC curve analysis demonstrated that the nomogram had a wide range of cut-off probabilities and showed excellent net benefits for threshold probabilities, indicating the potential clinical utility of the predictive model.

## DISCUSSION

The APACHE II scoring system has been widely used to measure severity of disease.^20^ It includes 12 points based on physiological parameters, age, and chronic health conditions, and has been applied to assess the severity and prognosis of critically ill patients with various diseases including heart, respiratory, and kidney disease.^22^–^24^ We choose the APACHE II score to evaluate the severity and prognosis of HS, which often involves damage to multiple systems.^14^ Previous research has shown that non-survivor HS patients had significantly higher APACHE II scores compared to survivors^25^–^27^ (10 points higher per Wei et al^14^). In our study, we observed that the median APACHE II scores of non-survivors were 13 points higher (*P*<0.001) than those of survivors, indicating a significant difference in disease severity between the two groups. However, when used alone, APACHE II scores may not be able to accurately predict the prognosis for patients with severe HS due to the complex nature of the syndrome involving multiple systems.^28^

Blood lactate levels serve as an indicator of reduced tissue perfusion and cellular hypoxia sensitivity.^29^ Elevated lactate levels are often seen in conditions with low perfusion, such as sepsis, shock, and trauma,^30^–^33^ as well as in HS patients,^34^–^36^ potentially due to hypoxia, ischemia, and hypermetabolism.^34^,^37^ In this study, the non-survival group had significantly higher lactate levels compared to the survival group. Many scholars have suggested that hypoalbuminemia can serve as an indicator of the severity of HS, but it is not directly associated with mortality.^38^ This study also found that there was no statistically significant difference in baseline albumin levels between the two groups of HS patients (*P*=0.126), which supports the previous findings. Additionally, the LAR has gained attention as a prognostic indicator in critically ill patients reflecting contrasting changes attributed to different mechanisms, with higher ratios indicating a more unfavorable prognosis.^39^–^42^ A higher LAR has been associated with a worsened prognosis in conditions such as sepsis, heart failure, acute pancreatitis, and cirrhosis.^43^–^46^

A study on pediatric patients with severe sepsis found that the predictive accuracy of the LAR was superior to that of the Lac clearance rate in determining the likelihood of developing multiple organ dysfunction syndrome (MODS) and mortality.^47^ The LAR demonstrated superior predictive ability compared to APACHE II in determining the occurrence of MODS and mortality during the early stages of ICU hospitalization of the septic patients.^48^ The LARs can be used as early prognostic markers for ICU patients with different initial lactate level and the presence of hepatic or renal dysfunction.^42^ It has also been used to predict short- and long-term mortality in critically HS patients,^45^ and it has been shown to be an excellent predictive value for myocardial injury in the elderly with severe community-acquired pneumonia.^33^ Thus, we chose the LAR as an indicator to evaluate the risk of 30-day death in HS patients in this study. And ROC curve analysis showed that it could be used to determine the prognosis of the disease. Overall, blood lactate levels and the LAR serve as important indicators for assessing disease severity and predicting prognosis in HS patients.

**Figure 5:**
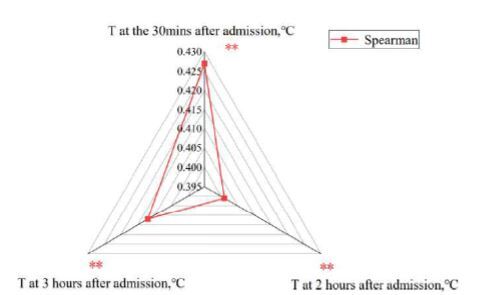
Correlation analysis of core temperature at different times after admission and heatstroke mortality. *T*, core temperature.

Some research has found that the mortality of HS is significantly affected by the degree and duration of high core temperature. The first 30 minutes after HS onset, also known as the “golden window,” is crucial for the outcome of HS.^16^, ^49^–^51^ Due to variations in the time from onset to hospital admission and potential inaccuracies in patient reports, we selected the core temperature at 30 minutes after admission as the golden window for HS in this study. This study demonstrated that the core temperature at 30 minutes, two hours, and three hours after admission all showed an association with HS mortality (Figure 5). Furthermore, the core temperature at 30 minutes after admission had the highest correlation with mortality, even after accounting for confounding factors. The higher the body temperature at the golden window correlated with the greater risk of death. Therefore, it was feasible to use the core temperature at 30 minutes after admission as an indicator to assess the risk of death. Armstrong and Casa have suggested that patients had a higher chance of survival if their core temperature was reduced to below 40.0°C within 30 minutes,^50^,^51^ while Heled indicated that it should be reduced to below 40.5°C.^49^ In China, expert consensus recommends reducing it to below 39.0°C within 30 minutes.^16^ In this study we found a lower risk of death when the core temperature at 30 minutes after admission was below 39.5°C.

In this study, the mortality rate of HS patients was 28.08%. The APACHE II score, the LAR, and the core temperature at 30 minutes after admission were found to be significant independent risk factors for mortality of HS patients. The higher the APACHE II score, LAR, and core temperature at 30 minutes after admission the higher the 30-day mortality rate will be in HS patients. These factors were used in a combined model for predicting 30-day mortality in HS patients. We conducted the ROC curve analysis to evaluate the predictive accuracy of the combined model and the three indicators used independently. The results showed that the predictive nomogram had a significantly higher AUC compared to the three indicators used independently, indicating better sensitivity and specificity of the predictive nomogram. We used the ROC curve and the calibration curve to verify the accuracy of the nomogram, and we also performed the decision curve analysis to evaluate the clinical usefulness of the nomogram.

The APACHE II score and LAR can be used in combination to evaluate the prognosis of other diseases such as septic shock, acute severe pancreatitis, heart failure, etc. We used core temperature at 30 minutes after admission as a quick preliminary indicator of HS for evaluating the treatment’s effectiveness within the initial 30 minutes, deliberately ignoring the comprehensive treatment. Overall, APACHE II score, LAR, and core temperature at 30 minutes after admission served as important independent risk factors for predicting 30-day mortality in HS patients. The combination of these factors in a predictive model can provide clinicians with valuable information in assessing the criticality of patients’ conditions and predicting mortality risk. Compared with the traditional scoring system, the new nomogram in this study improved the accuracy of the death risk assessment for HS. In clinical practice, the HS patient’s risk of mortality could be rapidly evaluated by this nomogram during early admission stages, which could help provide guidance for their subsequent clinical care.

As we mentioned previously, our study was not the first to use a nomogram in an attempt to predict the mortality of HS patients. Zhong et al^11^ indicated that the duration of cooling, HR at admission, and Sequential Organ Failure Assessment score are independent risk factors for death. However, it was important to note that the subjects of that study consisted solely of young adult males (19–27 years of age, mean 21).Wu et al^12^ confirmed that DIC, temperature, and GCS score were independent risk factors for death from exertional HS. The main subjects of the study were predominantly males (95.2%), and the possibility of selective bias also existed. Shao et al^13^ and Wei et al^14^ also constructed impressive nomograms for predicting survival in HS patients. Shao’s nomogram was based on WBC, Cr, ALT, maximum HR, invasive ventilation, and initial MAP and GCS score. Wei’s nomogram was based on neutrophil/lymphocyte ratio, platelet, troponin I, CK myocardial band, lactate dehydrogenase, human serum albumin, D-dimer, and APACHE-II scores. Both focused on elderly patients and paid no attention to the importance of early cooling treatment for the prognosis of HS.

Our nomogram has its own advantages. First, our study was conducted at two large, tertiary teaching hospitals, which made it possible for us to enroll more patients than in the previous studies. The gender and age distribution of our patients were more evenly distributed, and efforts were made to minimize the occurrence of selective bias. Then in addition to the APACHE II score, which is a classical mortality risk assessment system, we included two other indicators in the nomogram. The LAR is easily obtainable and can provide valuable information in evaluating the risk of HS-related mortality credited with different mechanisms. The core temperature at 30 minutes after admission considers both the severity of the patient’s condition at admission and the effect of cooling treatment on the prognosis. Thus, compared with previous research, this study provides a more objective and in-depth nomogram.

## LIMITATIONS

This study has several limitations that should be acknowledged. Firstly, the study was conducted in China, and HS was diagnosed according to the Chinese Expert Consensus on the Diagnosis and Treatment of HS. The following conditions were included in the diagnosis: the functional impairment of at least two of four organs (liver, kidney, striated muscle, and gastrointestinal tract); and severe coagulopathy or DIC, which are not included in the Bouchama HS criteria^52^ widely used in western countries. Secondly, due to sample loss and data loss, this study might not be sufficient to provide robust and reliable conclusions. Further research is needed to validate and refine the predictive model, including external validation in diverse healthcare settings in other countries.

## CONCLUSION

In this study we identified the APACHE II score, core temperature at 30 minutes after admission, and the lactate-to-albumin ratio as significant independent predictors of 30-day mortality in heatstroke patients. The combination of these three indicators demonstrated the best sensitivity and specificity in predicting mortality. Further research is required, specifically studies of different countries with larger sample sizes, to validate these results and enhance the accuracy of the predictive model. Considering that HS itself can cause multiorgan functional disturbance, we inferred that this conclusion might be applicable globally.

## Figures and Tables

**Table 1:** Characteristics between survivor and non-survivor groups of patients with heatstroke at admission.

	Total	Survivors	Non-survivors	P-value

	(N=203)	(n=146)	(n=57)	
Male	128(63.1%)	95(65.1%)	33(57.9%)	0.34
Female	75(36.9%)	51(34.9%)	24(42.1%)	
Age (years)	56.31±16.45	55.36±16.55	58.75±16.08	0.19
Classic Heat Stroke	102(50.25%)	66(45.21%)	36(63.16%)	0.02
Exertional Heat Stroke	101(49.75%)	80(54.79%)	21(36.84%)	
Time from onset to treatment (hours)	8.29(0.5,73)	9.11 (0.5,73)	6.19(1,24)	0.45
Underlying disease	131(64.53%)	92(63.01%)	39(68.42%)	0.47
Temperature at admission (°C)	39.09±1.77	38.61±1.55	40.32±1.69	<0.001
Heart rate (per minute)	118.47±31.51	111.92±28.95	135.25±31.81	<0.001
Mean arterial pressure (mmHg)	82.87±21.62	89.00±16.94	67.18±24.38	<0.001
Respiratory rate (per minute)	25.04±6.6	22.96±5.16	30.37±6.92	<0.001
Temperature at the 30 mintess after admission (°C)	38.77±1.73	38.28±1.45	40.02±1.77	<0.001
Temperature at 2 hours after admission (°C)	38.37±1.53	37.96±1.26	39.43±1.66	<0.001
Temperature at 3 hours after admission (°C)	37.97±1.36	37.57±1.07	38.99±1.50	<0.001
Cooling time (hours)	3.13(0,48)	3.17(0,48)	3.01(0,10)	0.15
White blood cell count (10^9^/ )	14.01(1.23,40.23)	13.56(3.49,36.57)	15.16(1.23,40.23)	0.06
Hemoglobin (g/L)	119.05(23,450)	119.79(66,181)	117.14(23,450)	0.12
Platelets (10^9^/L)	91.74(9,461)	88.68(9,461)	99.58(9,342)	0.61
Hs-CRP (mg/L)	38.57(0.1,200)	33.42(0.1,200)	51.76(0.5,200)	0.06
APTT (s)	49.55(18.4,183)	47.02(18.4,183)	56(29.2,106.1)	0.13
PT (s)	19.73(12,120)	18.02(12.5,90.2)	24.09(12,120)	0.21
Fibrinogen (g/L)	2.96(0.3,10.32)	2.69(0.3,6.14)	3.65(1.02,10.32)	0.05
D-dimer (ug/ml)	7.89(0.1,33)	7.17(0.1,33)	9.75(0.2,30)	0.98
Serum creatinine (umol/L)	158.39(1.36,1204.7)	128.96(12.78,1204.7)	233.76(1.36,894.5)	<0.001
Lactate (umol/L)	6.8(0.25,25)	4.72(0.25,25)	12.15(2.56,25)	<0.001
Blood glucose (mmol/L)	12.04(3.6,167)	10.54(3.6,36.7)	15.88(5.1,167)	0.09
AST (U/L)	249.04(13,3800)	230.95(13,3738)	295.39(19,3800)	0.70
ALT (U/L)	168.3(7,3628)	173.35(7,3628)	155.35(8,1561)	0.71
Albumin (g/L)	33.74(16.1,49.7)	34.01(16.1,44.2)	33.04(20.2,49.7)	0.13
CK (U/L)	737.99(12,15360)	803.06(12,15000)	571.34(12,15360)	0.78
Myoglobin (ng/mL)	1132.75(2.6,36125)	1115.06(2.6,36125)	1178.08(66,36024)	0.65
Troponin I (ng/L)	43.01(0.01,1001)	47(0.01,1000.1)	32.8(0.01,1001)	0.63
Lactate/albumin ratio (umol/g)	0.2(0.01,0.85)	0.13(0.01,0.73)	0.38(0.07,0.85)	<0.001
GCS score	8.07(3,15)	8.73(3,15)	6.37(3,15)	<0.001
APACHE II score	20.17(5,48)	16.46(5,37)	29.67(16,48)	<0.001
Hospital length of stay (days)	6.2(0.04,29)	6.82(0.29,27)	4.64(0.04,29)	0.06
Hospitalization fee (US dollars)	2,354(155,2,1706)	2,109(155,11,579)	3,116(305,21,706)	0.00

*Hs-CRP*, hypersensitive C-reactive protein; *APTT*, activated partial thromboplastin time; *PT*, prothrombin time; nine; *AST*, aspartate aminotransferase; *ALT*, alanine aminotransferase; *CK*, creatine kinase; *GCS*, Glasgow Coma Scale; *APACHE-II*, Acute Physiology and Chronic Health Evaluation.

**Table 2:** Risk factors for mortality of heatstroke patients in multivariable Cox regression model.

	HR	HR 95% CI	P-value
APACHE II score	1.102	1.067 ~ 1.139	<0.001
Lac/Alb ratio(umol/g)	12.772	3.934 ~ 41.466	<0.001
T at the 30mins after admission (°C)	1.639	1.338 ~ 2.007	<0.001

*APACHE-II*, Acute Physiology and Chronic Health Evaluation**;** *Lac/Alb*, lactate to albumin**;** *T*, core temperature.

**Table 3:** Logistic regression of APACHE II score, lactate-to-albumin ratio, and core temperature at 30 minutes after admission and the predictive model.

	AUC	Sensitivity	Specificity	Cut-off	P	95% CI
APACHE II score	0.867	0.965	0.616	18	0.000	0.817 – 0.916
Lac/Alb ratio (umol/g)	0.874	0.93	0.774	0.16	0.000	0.827 – 0.921
T at the 30 minutes after admission (°C)	0.774	0.702	0.788	39.5	0.000	0.694 – 0.854
The predictive model	0.928	0.825	0.918	−0.534	0.000	0.889 – 0.968

*APACHE-II*, Acute Physiology and Chronic Health Evaluation**;** *Lac/Alb*, lactate to albumin**;** *T*, core temperature.
